# Research on Lateral Resistance Performance of Prestressed Cross-Laminated Timber–Concrete Composite Structures under Reciprocating Loads

**DOI:** 10.3390/ma17112485

**Published:** 2024-05-21

**Authors:** Yong Xu, Xin Huang, Yingda Zhang, Yusen Qu, Yujie Fan, Guoqin Yang

**Affiliations:** School of Architecture and Civil Engineering, Xihua University, Chengdu 610039, China

**Keywords:** cross-laminated timber, concrete, prestressing, timber shear wall, reciprocating loading test, finite element analysis, structural analysis

## Abstract

Cross-Laminated Timber (CLT) and concrete composite structures represent an architectural system that integrates the strengths of both materials. In this innovative configuration, the CLT and concrete collaborate synergistically, harnessing their individual merits to achieve enhanced structural performance and functionality. Specifically, the CLT offers a lightweight design, superior bending resistance, and immense engineering plasticity, while concrete boasts exceptional compressive strength and durability. This study investigates the mechanical performance of CLT–concrete composite structures through quasi-static reciprocating loading tests in three full-scale CLT shear wall samples. Designed with varying initial prestressing forces and dimensions of the CLT panel, the prestressed CLT–concrete structures demonstrated a reduced dependence on the steel nodes, resulting in an increase in yield load, yield displacement, and maximum load-carrying capacity. Maximum capacity increased by 39.8% and 33.7% under initial prestressing forces of 23 kN and 46 kN on steel strands. Failure occurred due to localized compressive failure on prestressed steel strands and anchor plates. ABAQUS finite element analysis established three refined models, revealing that the increased initial prestressing force moderately enhanced stiffness but reduced ductility under similar cross-sectional dimensions. Furthermore, under consistent CLT material, dimensions, prestressing force, and loading conditions, prestressed CLT–concrete structures exhibited a higher maximum load-bearing capacity than prestressed CLT–steel composite structures. This study proposes structural design recommendations based on experimental and simulation results, incorporating specific assumptions.

## 1. Introduction

Cross-Laminated Timber (CLT) is a wood product composed of multiple layers of planks adhered together in an orthogonal manner using adhesives [1]. In comparison to lightweight timber structures using smaller section-sized timber for framing, CLT can be utilized in projects with larger cross-sectional dimensions to meet the demands of higher floors and greater spans in timber construction. In terms of mechanical performance, CLT exhibits higher rigidity, strength, and stability, making it suitable for applications on walls, floors, and roofs within buildings [2].

In recent years, there has been a significant amount of research on CLT structures [3]. Manalo and Aravinthan [4] designed three laminated timber specimens for shear and bending tests to investigate the impact of moisture content and density on the mechanical properties of the timber. The test results indicate that, while the moisture content and density have some influence on the mechanical properties of the specimens, the impact is not substantial. Additionally, there is almost no effect on the shear strength of the adhesive layers between the laminates.

Gong et al. [5] conducted a study on whether different structural characteristics affect the mechanical properties of CLT. Factors such as panel modulus, number of layers, panel thickness, and grain direction were considered. The analysis focused on the interlayer shear performance of Japanese larch CLT. The results indicate that the bending elastic modulus and strength of the Japanese larch CLT are influenced by the panel modulus and the number of layers, with less pronounced effects of the thickness and direction of the panel. The shear analogy method proves to be effective in predicting the equivalent shear stiffness of CLT.

Xie et al. [6] used hemlock as the material for CLT and conducted experimental research on the main strength direction of the CLT wall panels, as well as the shear strength and bending strength of the adhesive layers and interlayers for this tree species. The results of the study indicate that the perpendicular layer rolling shear strength of CLT is the main factor determining the bending strength and the interlayer shear strength of CLT.

Gavric et al. [7] conducted lateral cyclic loading tests on CLT walls using two construction methods: one using CLT as a whole panel and the other involving cutting the panel into smaller pieces connected by vertical connectors. Experimental analysis indicates that vertical connectors can enhance the energy dissipation capacity and ductility of the CLT wall system.

Hu and He [8] developed a CLT prestressed shear wall system consisting of CLT wall panels, prestressed tendons, and soft steel dampers. Using ABAQUS numerical simulation (version 6.14.4), they analyzed the effects of dampers and prestressed tendons on CLT shear walls. The results indicate that there exists an optimal combination of initial prestressing force in the tendons and material parameters, quantity, and shape of the dampers.

Sun et al. [9] designed four prestressed CLT shear walls with different initial prestressing forces of energy dissipation components and steel strands as influencing factors and subjected them to low-cycle reciprocating loading tests. The test results indicate that the CLT shear walls were generally intact after the experiments, with some deformation in the connectors. This suggests that prestressed CLT shear walls exhibit good lateral resistance performance. The position of the installation of the energy dissipation components has a certain influence on the energy dissipation capacity of the prestressed CLT shear walls.

From the above literature, it is evident that research on CLT structures is gradually shifting from node studies to structural investigations. Currently, the focus of CLT structure research lies primarily on timber–steel composite structures, with a predominant emphasis on metal connectors in node construction [10]. However, metal connectors face challenges such as corrosion, poor recoverability, and significant stiffness disparities [11]. In comparison, concrete exhibits strong plasticity and relatively small stiffness variations, making it a potential replacement for certain metal connectors. Taking into account the current status of CLT structure research and the drawbacks associated with timber–steel composite structures, this study proposes to investigate the lateral resistance performance of prestressed CLT–concrete composite structures using experimental research, theoretical analysis, and finite element simulations. This structural system can be efficiently and conveniently employed in a large-scale application for timber-framed houses.

## 2. Experimental Program

The experimental program of this research utilizes timber–concrete composite structures. While the majority of existing research on timber structures focuses on steel–timber instead of timber–concrete, the advantages of steel–timber composite structures include superior assemblability, faster construction speed, and a lighter overall weight compared to timber–concrete. However, steel–timber also possesses notable drawbacks such as higher cost, and the fire resistance, integrity, and seismic performance of concrete surpass steel. The primary factors contributing to the reduced load-bearing capacity of steel–timber structures are concentrated in the joint areas, where timber itself remains largely unharmed. Concrete can serve to replace part of the connectors, effectively enhancing the overall structural load-bearing capacity. As a result, the innovation of this study is the use of timber–concrete composite structures and the investigation of the overall structural load-bearing capacity.

### 2.1. Materials and Test Specimen

The CLT used in this research is Picea asperata–Pine–Abies with a moisture content of 12%. The prestressed steel is 1 × 7 non-bonded prestressed steel strands with a diameter of 15.2 mm. Additionally, rubber, splice steel plates, strain meters, markers, and strain gauges are utilized in this experimental program. The density of CLT is tested on 24 specimens according to Chinese standard GB/T1933-2009 [12]. Table 1 shows the dimensions and mass of specimens. After calculations, the average density of CLT in this study is 445.36 kg/mm^3^.

**Figure 1:**
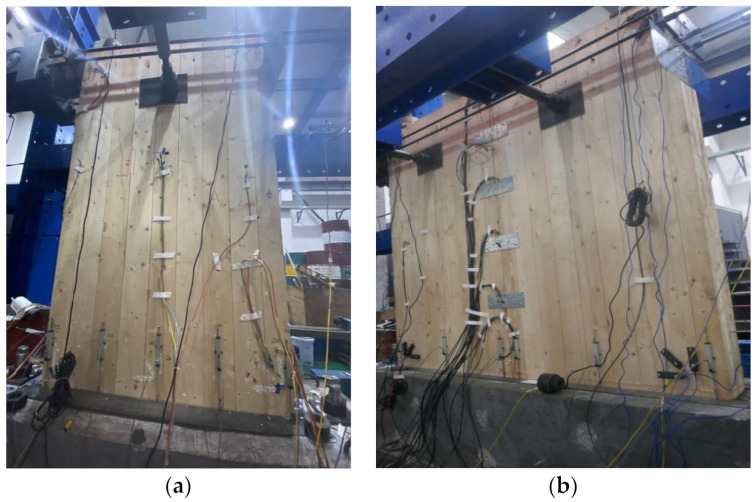
CLT test specimen: (**a**) side view; (**b**) front view.

This study investigates the lateral resistance performance of prestressed CLT–concrete composite structures under reciprocating loads. The actual specimen is depicted in Figure 1. Rubber is placed on both sides of the bottom groove of the concrete beam to prevent the wood from sagging under load. 

This experiment was designed with reference to the dimensions and assembly methods of CLT shear walls used in the experiments conducted by Sun et al. [9] and He et al. [13]. Note that in the experiments conducted for this study, prestress was employed to impart an axial force. It is noteworthy that the experimental specimens (PS, PH) referenced in the above studies were not subjected to any additional axial force. To ensure a more accurate comparison, the specimens in the present study were also not subjected to any additional axial force. Considering that the initial prestress ratio should be less than 50%, the initial prestress ratios for the three prestressed CLT single walls were set at 15.4%, 30.7%, and 15.4%, corresponding to the initial prestress forces in the steel strands of 23 kN, 46 kN, and 23 kN [9], respectively. Two single walls with the same dimensions but different prestressing forces were compared, and single walls with different prestressing forces were contrasted with different dimensions. Details of the specimen parameters including the dimensions of the specimens, the number of layers, and the thickness and diameter of the steel strand are listed in Table 2. The naming convention for the components is exemplified by the WSW-1-23 specimen, which signifies the first test specimen with an initial tension force of 23 kN applied to the steel strand. This notation will be consistently adhered to throughout the subsequent text, and thus, further elaboration will be omitted.

### 2.2. Layout of Measurement Points

**Figure 2:**
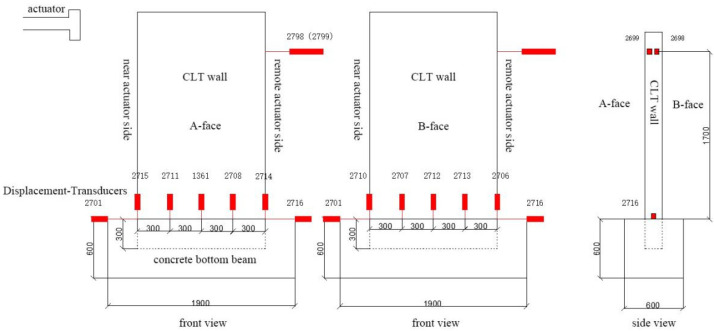
Schematic diagram of the displacement sensor arrangement.

Local displacement sensors were placed at a distance of 300 mm from the bottom surface of the CLT wall (at the junction of the wall and the concrete bottom beam) to measure the vertical displacement response of the bottom of the CLT wall. Two overall displacement sensors were, respectively, positioned on the side of the CLT wall near the actuator and on the side away from the actuator. Another two general displacement sensors were located on the side away from the actuator, 1700 mm from the upper surface of the concrete bottom beam (at the junction of the wall and the actuator), to measure the horizontal displacement response of the top of the CLT wall, as shown in Figure 2. 

**Figure 3:**
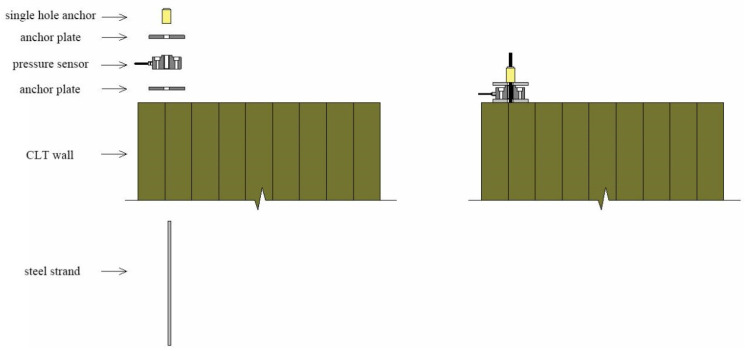
Schematic diagram of the connection arrangement.

The installation of prestressed steel strands requires the collective lifting of CLT wall panels along with the concrete bottom beam. The steel strands are inserted through the pre-reserved holes from the bottom, extending out from the top of the CLT wall panel. The tensioning end of the steel strands is equipped with a 175 mm × 175 mm × 12 mm anchor plate, a single-hole anchor, and a JLBU-1-50T pressure sensor (Bengbu, China). The connection method is illustrated in Figure 3.

**Figure 4:**
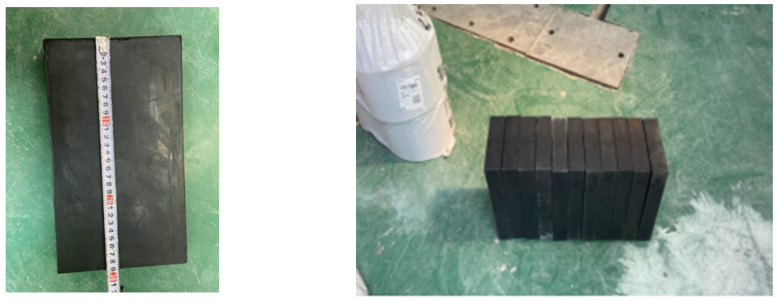
Actual photo of the rubber.

To protect the bottom and corners of the CLT wall panels from damage and enhance the overall energy dissipation capacity of the components, all three components require the use of rubber cushions with the same material and specifications, as illustrated in Figure 4. The bottom and both sides of the components are padded with rubber, with the dimensions and relevant parameters of the bottom rubber cushions detailed in Table 3. To prevent the side rubber cushions from being lifted during the reciprocating motion of the timber shear wall, a strong adhesive is applied to the inner walls of the corbels to securely adhere the rubber cushions.

**Figure 5:**
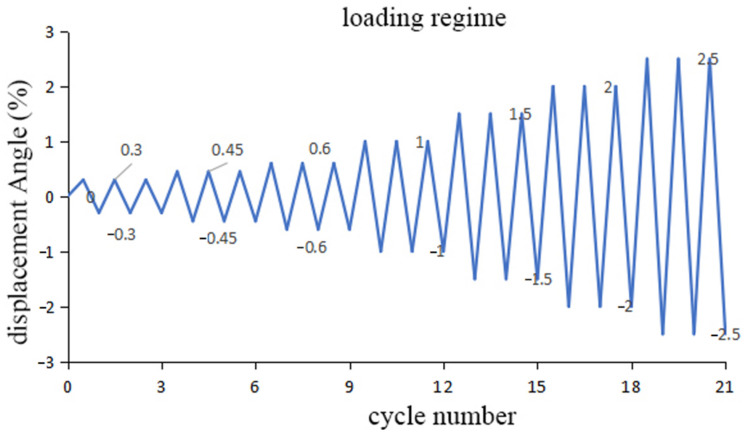
Loading regime for the experimental test.

The experimental program employed a suitable loading system for prestressed shear walls as per ITG-5.1-07 [14]. Since the maximum inter-story drift angle between floors for all similar systems should not exceed 2.5% [15], the target displacement angle during the lateral loading of the shear wall was set at 2.5%, corresponding to a target lateral displacement of 55 mm. Displacement-controlled loading was carried out with loading amplitudes of 0.3%, 0.45%, 0.6%, 1%, 1.5%, 2.0%, and 2.5%, with three cycles at each load level and a loading rate of 40 mm/min. The low-cycle reciprocating loading system is illustrated in Figure 5.

## 3. Test Results and Discussion

### 3.1. Test Results

**Figure 6:**
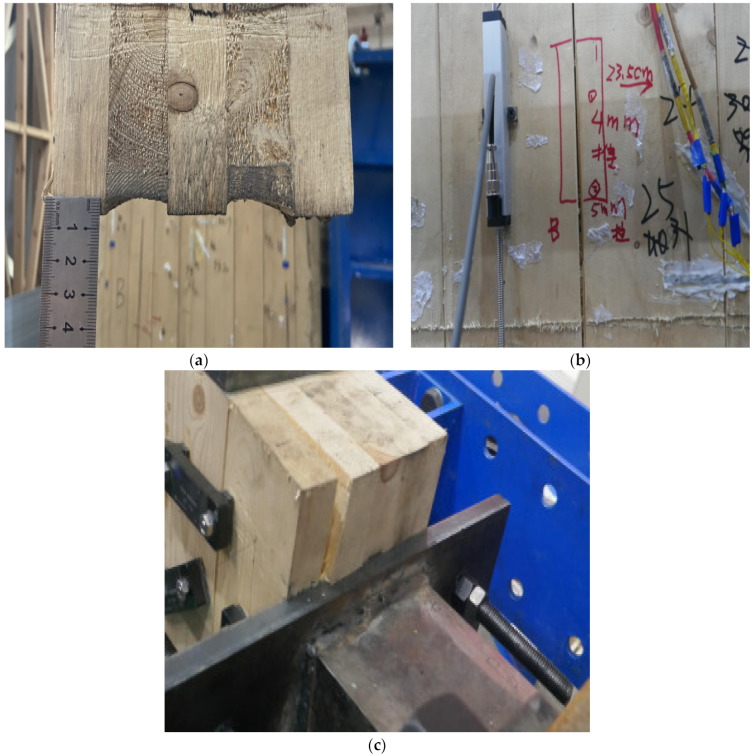
Experimental observations for (**a**) CLT cross-layers in WSW-1-23 subjected to compression; (**b**) increased gap in WSW-2-46; (**c**) separation of panels in WSW-3-23 due to eccentric compression.

The experiment evaluated various failure conditions that would cause the cessation of loading. These included the following scenarios: failure of the timber shear wall [16], leading to a loss of load-bearing capacity [17]; damage to the prestressed steel tendon system [18], preventing further loading; overrange conditions in the actuator, displacement sensors, and pressure sensors, rendering the test conditions invalid; and other failures preventing continued loading. Figure 6 illustrates the experimental observations for the three specimens.

On observation and analysis of the experimental phenomena of the three specimens, it can be found that the CLT shear walls in all three samples did not experience significant structural damage. In specimens WSW-1-23 and WSW-2-46, deformation of the CLT shear walls manifested mainly as cross-layer compression and increased panel gaps, as depicted in Figure 6a,b. In the case of specimen WSW-3-23, wall panel separation occurred at the actuator location due to eccentric pressure, resulting in adhesive surface separation. However, the panels themselves remained structurally intact, as shown in Figure 6c.

Numerous cracks appeared in the midspan and one-third of the span positions of the concrete bottom beam. This phenomenon was attributed to the absence of stirrups in these areas, which led to a weak overall tensile capacity. The uneven terrain at the testing site further contributed to this cracking [19].

All three specimens exhibited significant localized damage. The anchor plates in each specimen showed varying degrees of deformation, with specimen WSW-2-23 experiencing the most severe damage. This damage was attributed to stress concentration at the hole in the anchor plate and the limited contact area between the concrete bottom beam and the anchor plate [20].

### 3.2. Hysteresis and Skeleton Curves

**Figure 7:**
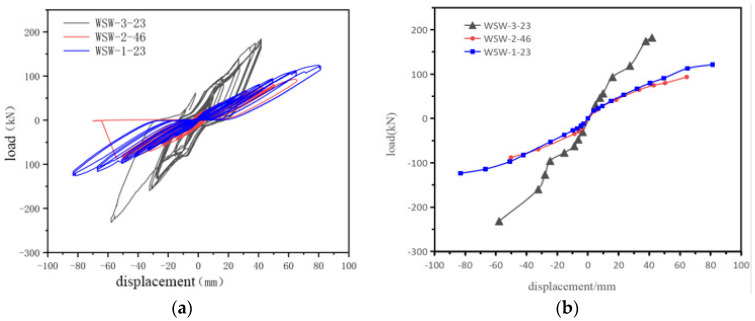
(**a**) Hysteresis curve and (**b**) skeleton curve for three specimens.

The hysteresis curves, skeleton curves, and low-cycle reciprocating load comparisons for the three specimens in this experiment are depicted in Figure 7. It can be observed that the hysteresis curves for all three specimens lack full rounding. Specimen WSW-1-23 exhibits a pronounced pinching phenomenon, attributed to the loss of initial tension in the prestressed steel strands during the test, preventing their contribution to self-centering in subsequent cycles. Specimen WSW-3-23 shows a relatively more rounded hysteresis curve, indicating that increasing the wall width effectively enhances the energy dissipation capacity.

It is also clear that higher initial prestressing forces lead to improved mechanical performance. Specimen WSW-2-46 consistently outperforms specimen WSW-1-23 in energy dissipation capacity during each loading cycle, due to its 46 kN initial prestressing force providing greater lateral stiffness and enhanced energy dissipation.

Moreover, larger aspect ratios contribute to better mechanical performance. Specimen WSW-3-23 consistently exhibits significantly better energy dissipation capacity than specimen WSW-1-23 in each loading cycle. The 1:1 aspect ratio results in larger deformations under the same displacement angle, providing a stronger energy dissipation capacity [21].

No descending branches are observed in the specimens. Skeleton curves reveal that none of the three specimens shows descending branches, indicating an absence of overall failure. This agrees with the experimental observation that despite significant localized damage, the walls did not undergo substantial damage.

### 3.3. Stiffness Degradation Curve

**Figure 8:**
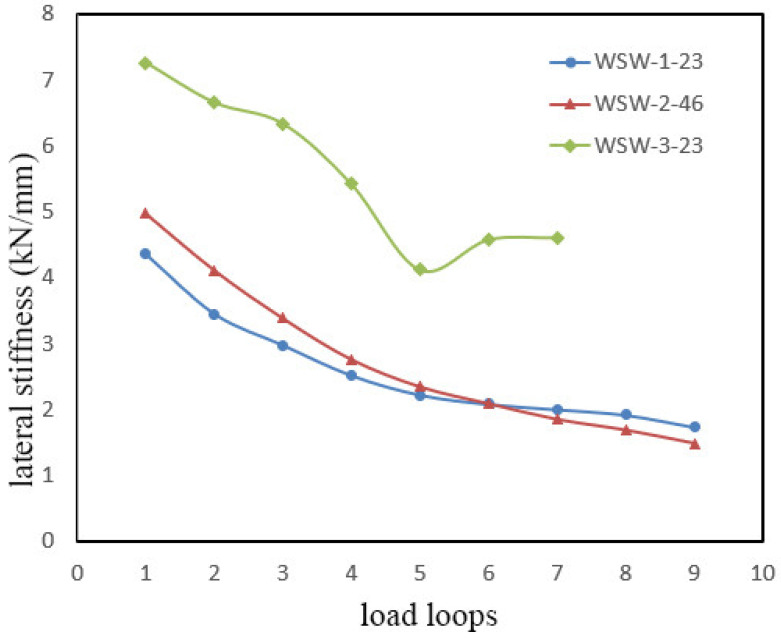
Stiffness degradation for three specimens.

The stiffness degradation curves for the specimens are presented in Figure 8, revealing that increasing the initial prestressing force in the steel strands contributes to a noticeable improvement in the stiffness of the specimen. Figure 8 illustrates that, with an increase in the initial prestressing force, the stiffness of the specimens improves significantly. However, specimen WSW-2-46 exhibits a slightly higher stiffness degradation rate than WSW-1-23. Until the sixth loading cycle, at a corresponding horizontal displacement of 42 mm, their stiffness remains approximately the same. Subsequently, WSW-2-46 experiences a gradual reduction in stiffness compared to WSW-1-23. This is attributed to the increasing internal force in the prestressed steel strands, causing compression deformation in the lower anchor plate of WSW-2-46, ultimately leading to failure. As a result, the reduction in elongation in the prestressed steel strands accelerates, resulting in a higher rate of stiffness reduction in WSW-2-46.

The aspect ratio significantly influences the stiffness of the specimen. As shown in Figure 8, the initial stiffness of specimen WSW-3-23 is 7.26 kN/mm, exceeding WSW-1-23 (4.36 kN/mm) and WSW-2-46 (4.9 kN/mm). This indicates that, under identical elastic modulus conditions, the aspect ratio plays a crucial role in the initial stiffness of CLT shear walls.

Unlike the stiffness degradation curves of the WSW-1-23 and WSW-2-46, which show a steep and gradual degradation, the curve for specimen WSW-3-23 features an additional rebound stage. The reverse occurs when the loading displacement reaches 42 mm, corresponding to the maximum internal force in the steel strands (163 kN), which does not reach the limit of the tensile capacity. At this point, severe bending in the fixture (loading steel plate) results in negative displacements during the sixth and seventh loading cycles, measuring 9.8 mm and 9.4 mm less than the design value. This discrepancy leads to a calculated displacement ratio that is larger than the actual value.

### 3.4. Energy Dissipation Analysis

**Figure 9:**
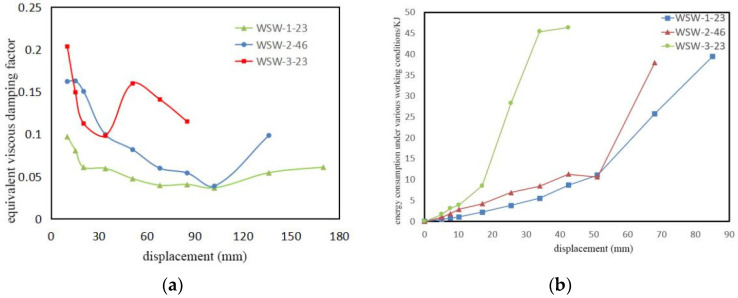
(**a**) Equivalent viscous damping coefficient and (**b**) energy dissipation capacity for three specimens.

Under the action of low-cycle reciprocating loads, the comparison of the equivalent viscous damping coefficient and energy dissipation capacity of the samples is shown in Figure 9. Analyzing Figure 9a reveals that, in the early stages of loading, the specimens rely mainly on the energy dissipation from compression and friction between the layers, as well as the shear and rupture of the connecting steel nails. As loading progresses and the deformations increase, the viscous damping coefficient shows a decreasing trend, resulting in an overall reduction in energy dissipation capacity. In the later stages of loading, the specimens dissipate energy through the elongation of prestressed steel strands (elastic stage) and deformation of the rubber on both sides of the concrete base beam (not reaching maximum deformation). Therefore, the energy dissipation capacity of the specimens exhibits a certain degree of recovery.

Analyzing Figure 9b shows that the energy consumption for each loading cycle of the three specimens exhibits a continuous increase, with none reaching the yield displacement. Enhancing the initial prestressing force in the steel strands contributes to improved energy dissipation capacity in the specimens. In Figure 9b, the curve for WSW-2-46 shows a greater energy dissipation capacity compared to WSW-1-23 under the same displacement control until the eighth loading level (lateral horizontal displacement of 52.5 mm), with a maximum difference of 2.9158 kJ. However, beyond the seventh loading level, the curve for WSW-2-46 shows a decreasing trend. This is due to the stress concentration on the anchor plate of the prestressed steel strands, leading to uneven stress distribution and reduced stress in areas other than the compressed region. The inward concavity of the anchor plate results in insufficient elongation of the prestressed steel strands and decreased energy dissipation capacity. During the ninth negative loading level, the energy dissipation capacity increases as the anchor plate on the side away from the actuator is damaged.

Moreover, the aspect ratio significantly influences the dissipation of the energy from the samples. The curve corresponding to WSW-3-23 shows significantly higher energy dissipation capacity than the other two specimens. The maximum difference in energy dissipation capacity between WSW-3-23 and WSW-1-23 is 39.8 kJ, and with WSW-2-46, it is 36.93 kJ. Additionally, after completing the third loading level, the energy dissipation capacity of WSW-3-23 increases sharply. This is attributed to the greater deformation of the prestressed steel strands and rubber under the same displacement control. However, after the sixth loading level, the curve exhibits a decreasing trend, indicating a reduction in the energy dissipation capacity. This is because during the seventh loading level (horizontal lateral displacement of 84 mm), the loading fixture (steel plate) experiences severe bending, preventing the specimen from reaching the design displacement during negative loading.

### 3.5. Prestress Loss Analysis

**Figure 10:**
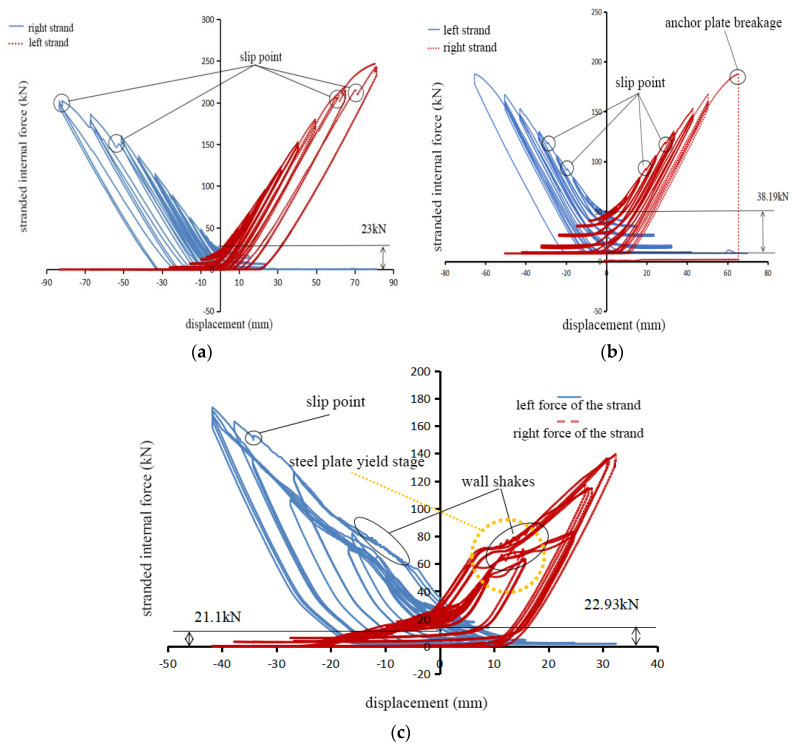
Internal forces of the steel strand for (**a**) WSW-1-23; (**b**) WSW-2-46; and (**c**) WSW-3-23.

In order to investigate the variation in internal forces of the steel strands, data with an inter-story displacement angle of 2.5% were used for analysis in all three specimens. The curves that depict the changes in the internal forces of the steel strands for each sample are shown in Figure 10.

Analysis of the steel strand force variation reveals that when loaded to an interlayer displacement angle of 2.5%, the residual tension of a single steel strand in the three specimens is close to zero, indicating that the steel strands are essentially no longer functioning at this point.

The magnitude of the initial tension significantly influences the tension loss of the steel strands. In Figure 10a,b, when the WSW-2-46 sample reaches the target displacement angle of 2.5%, the loss-to-initial-tension ratio for the left and right steel strands is 0.42 and 0.44, respectively. For the WSW-1-23 specimen, the ratio for both steel strands is 0.99. This indicates that as the initial tension increases, the ratio of tension loss to initial tension decreases.

The force curves of the steel strands in all three specimens exhibit slipping. This is due to a 2 mm installation gap between the CLT shear walls and the rubber on both sides. Therefore, when the maximum static friction between the bottom surface of the wall panel and the concrete bottom beam is insufficient to resist horizontal shear, the wall panel will experience a slight horizontal slip, resulting in a sudden drop in tension in the steel strands.

The force curve of the steel strands in the WSW-3-23 specimen appears slightly irregular. As observed in Figure 10c, the curve exhibits a point of oscillation, attributed to the fact that this specimen was assembled using a joint method. During the experiment, when the friction force and shear strength of the steel nails at the joint of the two wall panels are insufficient to counteract the increased tension in the prestressed steel strands due to elongation, the wall panels will experience displacement. At the moment of displacement, the wall exhibits a noticeable vibration, causing oscillations in the tension of the steel strands. As the lateral displacement increases, the degree of displacement becomes more pronounced [22].

### 3.6. Analysis of Actual Strain for CLT Shear Walls

**Figure 11:**
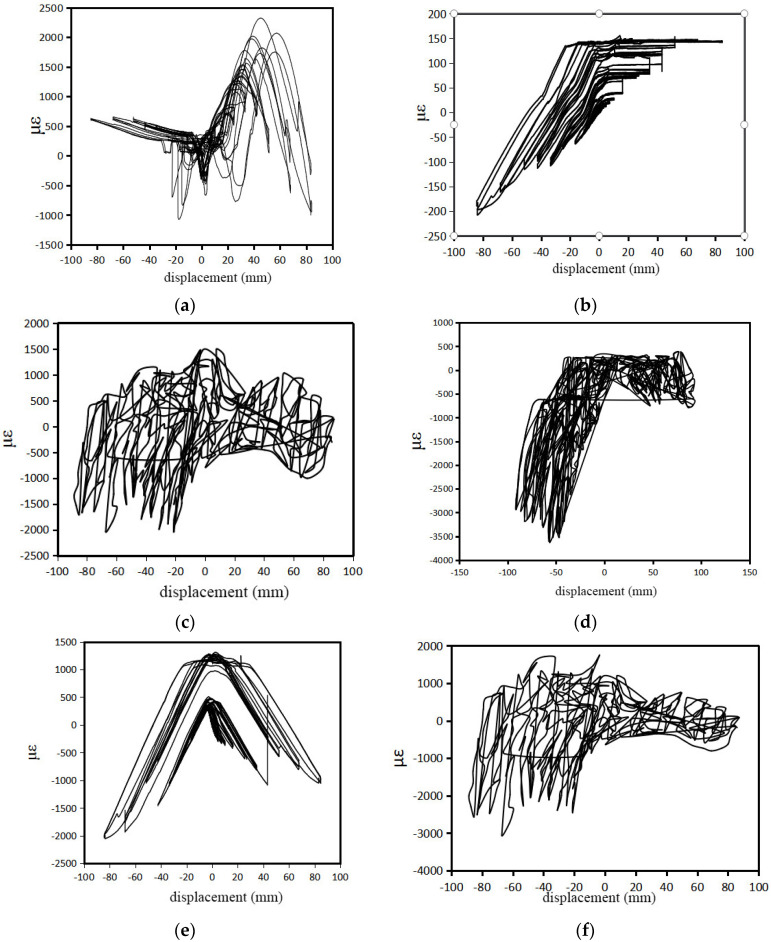
(**a**) Horizontal wall strain in the upper part of WSW-1-23; (**b**) vertical wall strain in the upper part of WSW-1-23; (**c**) horizontal wall strain in the bottom of WSW-1-23; (**d**) vertical wall strain in the bottom of WSW-1-23; (**e**) steel strand strain in the location corresponding to the placement of prestressed steel strands in WSW-1-23; (**f**) wall strain in the upper corner of the WSW-3-23 specimen.

The strain diagrams for each sample are presented in Figure 11, and the analysis reveals that in the horizontal direction near the actuator side of the upper part of the CLT shear wall (Figure 11a), both tensile and compressive strains are observed during reverse loading in the WSW-1-23 specimen. The strain patterns are similar, with relatively large tensile strain values.

As shown in Figure 11b, both the tensile and compressive deformations are presented in the vertical direction. During forward loading, compressive strains occur due to the elongation of prestressed steel strands. In reverse loading, tensile deformations are observed, restricted by the rotation of the CLT shear wall and compression deformation of the bottom rubber pads.

Figure 11c represents the horizontal strains at the bottom of the CLT shear wall. It shows slight tensile deformations during small displacements in forward loading and compressive deformations during large displacements. Reverse loading mainly exhibits tensile strains, limited by the lateral rubber pads. Figure 11d depicts compressive strains encountered during loading, wherein significant compressive strain values approaching approximately 3500 με are observed. This exceeds the elastic limit strain of CLT under cross-grain compression, resulting in considerable deformation. The peak compressive strain aligns with a vertical distortion that concurs with the measured transverse compressive deformation post-experimentation.

Figure 11e represents the deformation in the position of the prestressed steel strands in the CLT shear wall. It shows tensile strains before 20 mm and compressive strains after surpassing 20 mm in both forward and reverse loading. The component tends to self-reset, demonstrating self-resetting performance. Figure 11f represents the strain in the upper corner near the actuator side of the WSW-3-23 specimen. It reveals a yielding phenomenon with a significant increase in strain and a corresponding small increase in displacement. The maximum compressive strain reaches 2500 με, consistent with the experimental observations.

### 3.7. Comparison with Existing Literature

**Figure 12:**
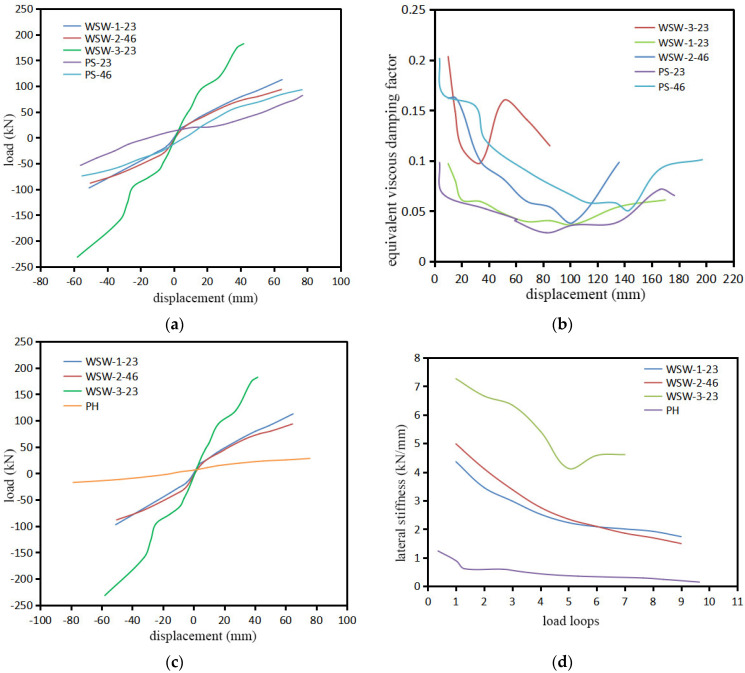
Comparisons of experimental results for (**a**) load compared with PS; (**b**) equivalent viscous damping coefficient; (**c**) load compared with PH; (**d**) lateral stiffness.

The experimental specimen consists primarily of CLT shear walls, prestressed steel strand systems, and a concrete bottom beam. The materials and loading systems used in this experiment are consistent with those in Refs. [6,8]. Therefore, for comparison purposes, the samples in Ref. [6] are denoted as PS, those in Ref. [8] as PH, and the samples in this study as PQ, as shown in Table 4 and Figure 12.

From Figure 12, it can be observed that the stiffness of the WSW-1-23 and WSW-2-46 samples is slightly higher than that of the PS-23 and PS-46 samples under the same displacement control. The energy dissipation capacity of the PS-23 sample is nearly equal to that of the WSW-1-23 sample, but the energy dissipation capacity of the PS-46 sample is slightly higher than that of the WSW-2-46 sample. This is because during the test loading process, the bottom anchor plate of the WSW-2-46 sample experienced compressive deformation, leading to a decrease in the total energy dissipation capacity. The stiffness of the WSW-1-23 and WSW-2-46 samples increased by 71.1% and 74.2%, respectively, compared to the PH sample, demonstrating that prestressed steel strands significantly improve the stiffness of the CLT shear walls. The stiffness of the WSW-3-23 specimen increased by 83.3% compared to the PH specimen.

In Table 4, it can be observed that, compared to PS samples, PQ samples, with the same initial tension force on steel strands, use a steel-reinforced concrete base beam instead of a steel bottom beam. When the initial tension force in the steel strands is 23 kN, the maximum load-carrying capacity of the PS-23 specimen is 52.5 kN, while the PQ-23 specimen achieves a maximum load-carrying capacity of 73.4 kN. The maximum load-carrying capacity of the PQ specimen is increased by 20.9 kN, representing a 39.8% improvement over the PS specimen. When the initial tension force on the steel strands is 46 kN, the maximum load-carrying capacity of the PS-46 sample is 60.8 kN, while the PQ-46 sample achieves a maximum load-carrying capacity of 81.3 kN. The maximum load-carrying capacity of the PQ specimen is increased by 20.5 kN, representing a 33.7% improvement over the PS specimen.

Based on the findings, it is evident that the application of WSW significantly enhances the stiffness and energy dissipation capacity of CTL shear walls compared to those without prestress (PH). Furthermore, the combination of reinforced concrete bottom beams with rubber-restrained glue-laminated timber walls (WSW) exhibits a certain degree of improvement in the maximum load-bearing capacity of CLT shear walls compared to steel bottom beams paired with angle steel-restrained walls (PS).

## 4. Numerical Simulations

### 4.1. Finite Element Model

To perform a material parameter analysis of the specimens, a finite element model was established through simulation to replicate experimental observations. The material parameter values used in this experiment were identical to those specified in the material property parameters of Ref. [23], as the latter employed the same tree species and manufacturer as the present study. Regarding the failure criterion, the mechanical properties of wood are significantly influenced by its moisture content, and its plastic deformation is associated with hydrostatic pressure, rendering its yield criterion rather complex. In this paper, the yield criterion is based on the Hill theory [24], which is widely adopted for most wood materials. Specifically, it extends the von-Mises theory for isotropic materials to anisotropic materials.

In the model, the prestressed steel strands have specifications of 1 × 7, a diameter of 15.2 mm, a nominal area of 139 mm^2^, an elastic modulus of 1.95 × 105 N/mm^2^, and a Poisson’s ratio of 0.25. The rubber material has a hardness of 60, an elastic modulus of 445 N/cm^2^, a shear modulus of 106 N/cm^2^, a correction factor of 0.57, and a Poisson’s ratio of 0.4. Note that the influence of elastic properties of materials on non-homogeneous structures and their determination play an important role in the estimation of the structural response of heterogeneous materials such as CLT [25,26]. In this study, the elastic properties are obtained based on Ref. [23] because the materials used in this study are identical to Ref. [23]; information on the elastic properties is provided in Table 5. In Table 5. E_L_, E_R_, and E_T_ represent the elastic moduli in the longitudinal, radial, and tangential directions of the CLT grain, respectively. G_LR_, G_LT_, and G_RT_ denote the shear moduli for the cross-grain, radial, and tangential sections of the wood, whereas γ_LR_, γ_TR_, and γ_LT_ signify the Poisson’s ratios for the corresponding cross-grain, radial, and tangential sections. 

As evident from the skeleton curve, variations in the initial tensile force do not significantly impact the elastic modulus, whereas dimensional changes have a significant influence on it. Under the experimental conditions of this study, the shear wall exhibited minimal deformation and did not suffer any damage, thus justifying its consideration as a rigid body.

**Figure 13:**
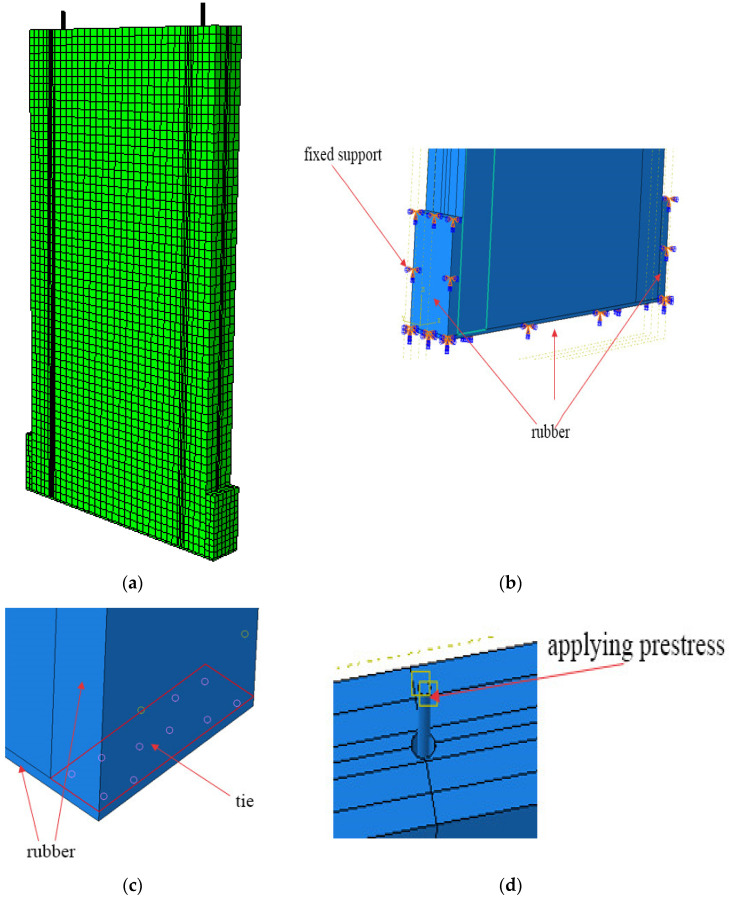
Details of the FEM: (**a**) mesh; (**b**) boundary condition; (**c**) contact; (**d**) initial prestressing force.

Due to the regularity of the model, the meshing was applied directly to various components through a consistent mesh size, and we ultimately selected a 20 mm division, as shown in Figure 13a. By comparing the initial and final states of the CLT before and after the test, it was observed that there was almost no sliding, splitting, or detachment between the CLT layers. Therefore, the contact between the layers was defined as a tied contact. Similarly, no separation was observed between the rubber on both sides and the rubber interface; hence, this contact area was also defined as a tie contact. During the test, there was no slippage in the concrete base, so a fixed support was applied to the model base. In this model, the impact of gaps between layers was not considered [27]. The boundary conditions and contacts of the model are shown in Figure 11c and Figure 13b. This study utilized a temperature reduction method to apply prestress to the steel strands, and the loading procedure matched the actual experiment (see Figure 13d) [28].

### 4.2. Finite Element Analysis

#### 4.2.1. Stress Analysis

**Figure 14:**
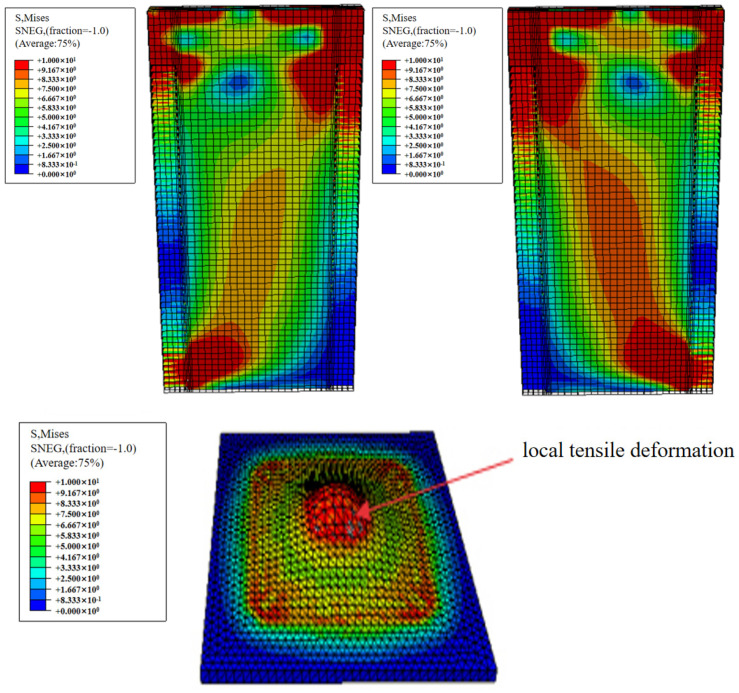
Stress diagram of each component of the WSW-1-23 specimen.

Taking the WSW-1-23 specimen as an example, the finite element analysis results are presented in Figure 14. It can be observed that, under the action of horizontal low-cycle reciprocating loads, the finite element model essentially captures the stress characteristics of the shear wall, with the maximum stress initially occurring in the wall panel. As displacement increases, the internal forces in the prestressed steel strands increase, leading to increased stress in the contact area between the anchor plate and the wall. Overall, there is no significant general deformation in the wall itself, and the component deformation is concentrated primarily in the anchor plate, consistent with the experimental process.

The finite element analysis models of the WSW-1-23 and WSW-2-46 specimens reveal that under the influence of low-cycle reciprocating loads, significant stresses occur at the actuator position and on both sides of the CLT wall panel at the bottom. From the contour plots, it is evident that the stress at the actuator position of WSW-2-46 is greater than that of WSW-1-23. This is attributed to the slightly higher initial prestress in the steel strands of WSW-2-46, resulting in a higher initial stiffness compared to WSW-1-23. Over time, as the rotation angle of the CLT wall panel gradually increases, the deformability of the rubber at the bottom and on both sides decreases, and the hardness increases. Consequently, the stress at these locations on the CLT wall panel gradually increases, although it remains relatively smaller compared to the stress at the actuator position. In general, the finite element simulation results align with the loading conditions experienced by the CLT wall panel during the experimental process.

#### 4.2.2. Comparison of Skeleton Curves

**Figure 15:**
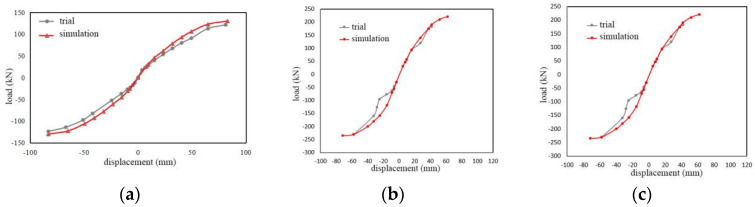
Comparisons of skeleton curves for (**a**) WSW-1-23; (**b**) WSW-2-46; (**c**) WSW-3-23.

The comparison between the ABAQUS finite element simulation skeleton curve and the experimental test skeleton curve is illustrated in Figure 15. It can be seen in Figure 15 that the experimental and numerical analysis skeleton curves align well. With the exception of a few points where the error is relatively large, the remaining errors are all within 5%. Instances with significant errors occur for WSW-2-46 and WSW-3-23 samples when loaded to −20 cm. This discrepancy is attributed to the significant deformation that occurred in the Q235 steel anchor plate during actual loading at this stage. In conclusion, the results of the numerical analysis exhibit good agreement with the experimental results in terms of the loading process and skeleton curves, supporting the consideration of this numerical model for the analysis of material parameters.

### 4.3. Parameter Analysis

#### 4.3.1. Initial Prestressing Force of the Steel Strand

**Figure 16:**
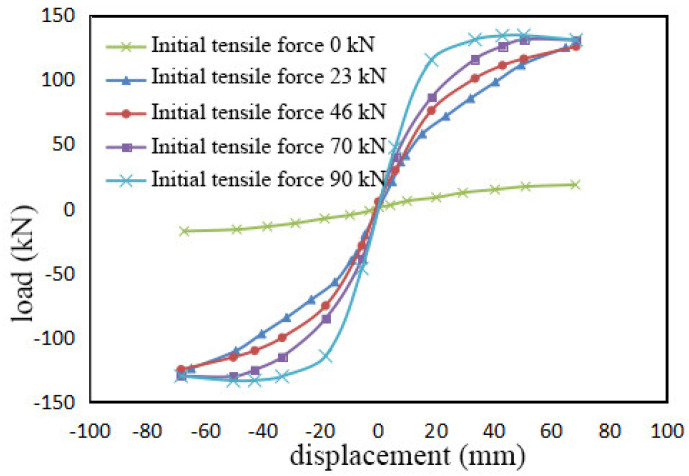
Skeleton curves under different initial prestresses.

Using the finite element model of the WSW-1-23 specimen as a reference, different loading conditions were simulated under varying initial tensions by adjusting the cooling temperature difference of the prestressed steel strands in the predefined field. The simulation results are presented in Figure 16. It can be seen in Figure 16 that when the initial tension is 0 kN, the sample is almost unburdened. The skeleton curve at this point is nearly a line with a very small slope, indicating poor load-bearing performance. This phenomenon underscores the indispensability of prestressed steel strands in the load-bearing capacity of the composite structure.

As the initial tension increases, the initial stiffness of the specimen improves significantly. With the growth of the initial tension, the internal forces in the steel strands increase, thus enhancing the initial stiffness of the specimen by increasing its resistance to horizontal lateral displacement moments.

The ultimate bearing capacity of the specimen shows little change with variations in initial tension. In the later stages of loading, as the initial tension diminishes, the steel strands almost cease to function, resulting in an almost unchanged final bearing capacity.

#### 4.3.2. Aspect Ratio

**Figure 17:**
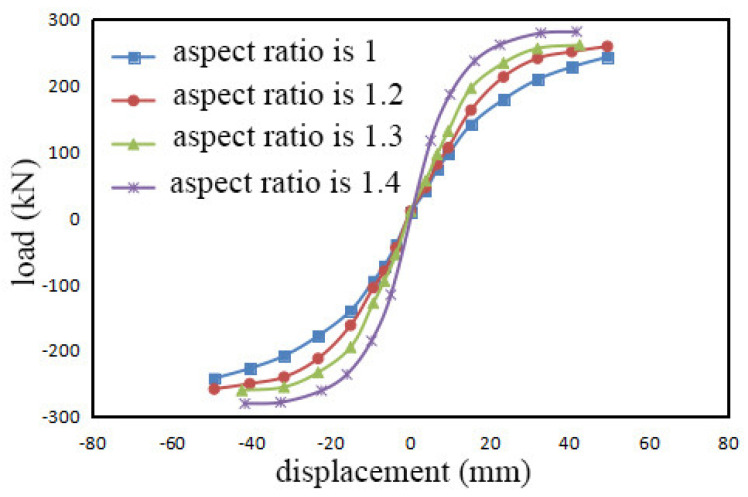
Skeleton curves at different aspect ratios.

Taking into account the practical requirements of building module proportions, simulations were performed for aspect ratios of 1.2, 1.3, and 1.4, and the simulation results of finite elements are presented in Figure 17. It can be seen that under the same initial tension conditions, the aspect ratio of the component has some influence, but the impact is relatively small. As the aspect ratio increases, there is little change in the bearing capacity of various load levels. The maximum bearing capacity of simulations with aspect ratios of 1.2 to 1.4 increases by 6.2%, 6.9%, and 8.0%, respectively, compared to the simulation result with an aspect ratio of 1.

With an increase in aspect ratio, the elastic–plastic stage displacement of the structure gradually decreases. This is because with a larger aspect ratio under the same lateral displacement control, the rotation angle increases. As a result, the corner of the wall rises to a higher height under the same lateral displacement conditions, leading to a potential increase in the elongation and internal forces of the steel strands, which may cause excessive local compression at the top of the CLT wall panel, resulting in bulging or splitting and separation between the layers.

Increasing the aspect ratio also leads to a longer support length of the wall and floor, making it prone to out-of-plane instability. In addition, it causes a significant concentration of stress in the anchor plate, resulting in a decrease in the overall load-bearing capacity of the component.

## 5. Analytical Calculations

### 5.1. Bearing Capacity of Overall Structure

According to the Chinese Standard GB50005-2017 [29], the bearing capacity of timber structures for compressed bending members and eccentrically compressed members is calculated using Equation (1).

(1) $$\frac{N}{A_{n}f_{c}}+\frac{M_{0}+Ne_{0}}{W_{n}f_{m}}\leq 1$$

where N is the axial compressive design value; $A_{n}$ and $W_{n}$ represent the net cross-sectional area and the net cross-sectional resistance moment of the member, respectively; $f_{c}$ and $f_{m}$ are the design values of the compressive strength and the bending strength in the longitudinal grain direction, respectively; $e_{0}$ is the initial eccentricity of the axial pressure on the member (in mm); $M_{0}$ is the design value of the maximum initial bending moment at the midspan under lateral load, and can be determined using Equation (2).

(2) $$M_{0}=F\cdot h+M$$

where F is the design value of lateral load; h is the height of the specimen; M is the initial moment.

**Figure 18:**
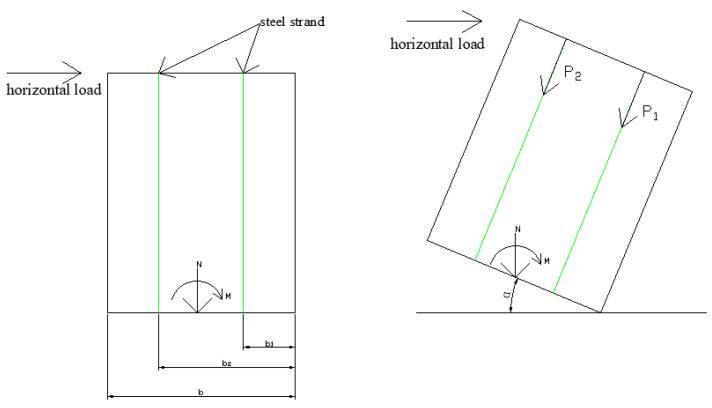
Force decomposition diagram.

In Equation (1), the axial pressure is provided by the steel strands. As the horizontal load increases, the elongation of the steel strands and the internal force gradually increase. Therefore, the value of axial compressive force in the equation undergoes a changing state and its force diagram is shown in Figure 18. In order to further derive the strength verification formula that fits the current experiment, two assumptions are made in this paper. First, the steel strands always remain straight without deformation by bending. Second, the internal force of the steel strands does not exceed the maximum bearing capacity, and there is no change in the cross-sectional area before the maximum bearing capacity is reached.

Under these assumptions, the axial pressure value for a single steel tendon is given by Equation (3):

(3) $$N_{1}=P\cos\alpha =f_{ptk}A\cos\alpha$$

where $N_{1}$ is the maximum axial pressure of a single steel tendon; P is the internal force of the steel tendon; $f_{ptk}$ is the ultimate strength standard value of the seven-strand steel tendon; A is the cross-sectional area of the seven-strand steel tendon; and α is the rotation angle.

Subsequently, the axial pressure design value for multiple steel strands can be calculated using Equation (4):

(4) $$\sum N=\frac{\sum b_{n}}{b_{max}}P=\frac{\sum b_{n}}{b_{max}}f_{ptk}A\cos\alpha$$

where $b_{max}$ is the maximum horizontal distance between the location of the nth steel tendon and the base point of the CLT wall panel; $b_{n}$ is the horizontal distance between the location of the nth steel tendon and the base point of the CLT wall panel.

Substituting Equation (4) for the axial pressure of the steel strands in Equation (1) and considering small deformations where cos⁡α is approximately equal to 1, the strength verification equation for prestressed CLT wall panels can be derived as Equation (5):

(5) $$\frac{\frac{\sum b_{n}}{b_{1}}f_{ptk}A}{A_{n}f_{c}}+\frac{M_{0}+\frac{\sum b_{n}}{b_{1}}f_{ptk}Ae_{0}}{W_{n}f_{m}}\leq 1$$

Through the above equation, the strength verification is conducted for the three specimens in this study and the finite element model. The experimental data and finite element simulation output data are separately entered into Equation (5) for error analysis, and the results are shown in Table 6. From Table 6, it can be observed that the strength verification values are relatively small, indicating that the samples did not experience overall failure. This conclusion aligns with the experimental observation that the specimens did not experience overall wall failure and the failures were mainly concentrated in steel strand failure or reaching maximum bearing capacity, bottom anchor plate failure, and concrete failure. 

For the WSW-1-23 specimen, the errors between the experimental, simulated, and theoretical values are 7.5% and 5.6%, respectively, within an acceptable range. For the WSW-2-46 sample, the error between theoretical and experimental values is 23%. This is attributed to the stress concentration at the bottom anchor plate of the steel strands during loading, causing the anchor plate to protrude under compression until failure. This leads to a decrease in the elongation of the steel strand and the internal force under the same control of lateral displacement, resulting in a large deviation between experimental and theoretical values. For the WSW-3-23 specimen, the errors between experimental, simulated, and theoretical values are 8.3% and 4.8%, respectively. The cause of the deviation is that this sample is made up of two boards with dimensions of 1200 × 2400 × 175, and a relative displacement occurs between the boards during the low-cycle reciprocal loading.

Based on the error analysis of experimental observations, finite element simulation, and theoretical values, it can be concluded that Equation (5) applies to the general strength verification of the specimens described in this study. Combined with experimental observations and finite element simulations, it is evident that prestressed CLT–concrete shear walls do not undergo overall failure and the shear walls can be treated as rigid bodies. The failure of the samples is characterized mainly by rupture of the steel strand, failure of the anchor plate, and failure of the concrete bottom beam. Under external loads, the specimens rotate around the rigid body foothold, requiring a separate analysis of the three situations mentioned above.

### 5.2. Bearing Capacity of Steel Strand (F_1_)

When subjected to horizontal loads, the bottom corners of the CLT shear walls undergo rotation, resulting in the elongation of the steel strands. For this structure, failure of the steel strands would lead to loss of structural load-bearing capacity, necessitating analysis.

The maximum tensile forces on the steel strands for the specimens WSW-1-23, WSW-2-46, and WSW-3-23 are 246.5 kN, 227.4 kN, and 229.38 kN, respectively. These values are all less than, but close to, the maximum bearing capacity of the steel strands, which is 258.54 kN. Due to the significant risk of rupture of the steel strands, it is determined that the component loses load-bearing capacity when the internal force of the steel strands approaches the maximum bearing capacity.

The equation to calculate the bearing capacity of the steel strand (*F*_1_) is as follows:

(6) $$F_{1}=f_{ptk}A$$

### 5.3. Local Compressive-Bearing Capacity of Concrete (F_2_)

**Figure 19:**
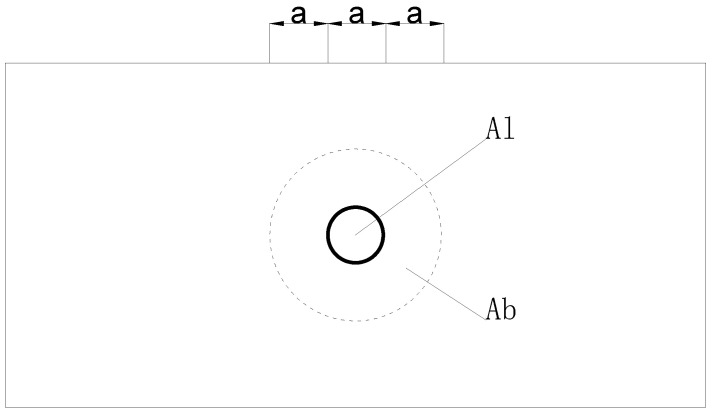
Bottom area under local compression.

Under horizontal loading, there is localized contact between the CLT shear wall and the concrete. When localized concrete failure occurs, the CLT shear wall will lose its load-bearing capacity. According to the Chinese Standard JTG 3362-2018 [30], the cross-sectional dimensions of the locally compressed zone in the reinforced concrete components should satisfy Equation (7), with the calculation results shown in Table 7. Through the calculation, it is determined that the locally compressed concrete zone meets the requirements.

(7) $$F_{2}\leq \frac{1.3\eta_{s}\beta f_{c}A_{ln}}{\gamma_{0}}$$

where $\eta_{s}$ is the local compression correction factor for concrete; β is the local compression strength enhancement factor for concrete and can be calculated using Equation (8).

(8) $$\beta =\sqrt{\frac{A_{b}}{A_{l}}}$$

where $A_{b}$ is the calculated bottom area under local compression, as shown in Figure 19; $A_{ln}$ and $A_{l}$ are the local compressed areas with and without deducting the hole, respectively.

### 5.4. Bearing Capacity of Anchor Plates (F3)

**Figure 20:**
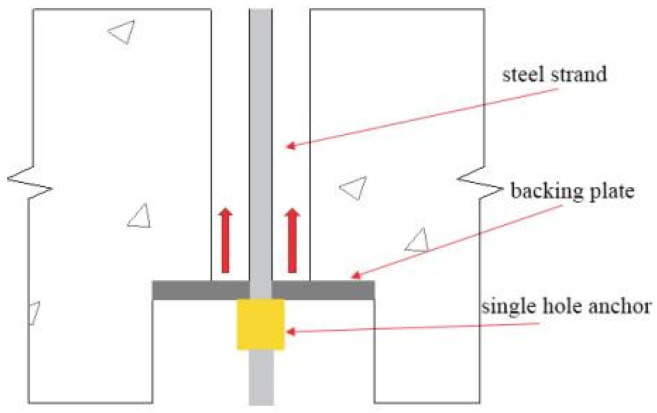
Concrete bottom beam anchor zone.

In non-bonded prestressed structures, the commonly used anchorage system is the basic anchor plate, with many instances utilizing a single-hole anchor plate [31]. The CEC 180 prestressing construction code specifies a plate thickness of 14 mm; however, in practical applications, a significant number of plates with a thickness of 12 mm are observed [32]. Given the crucial role of the plate in the bearing capacity in this experiment, a calculation and analysis of the local compressive bearing capacity of the plate are conducted. As a result, the different thicknesses of anchor plate are presented in Table 8. This analysis provides insights for optimizing the structure in subsequent phases. The anchorage zone is illustrated in Figure 20.

According to the AASHTO specifications [32], the anchor plate can be verified as rigid or flexible using Equations (9)–(11).

(9) $$\frac{n}{t}\leq 0.08\sqrt[{3}]{\frac{E_{s}}{\sigma_{1n}}}$$

(10) $$\sigma_{1n}=\frac{0.8f_{ptk}A_{p}}{A_{1n}}$$

(11) $$F_{3}=\sigma_{1n}\cdot A_{d}$$

where $E_{s}$ is the modulus of elasticity of the anchor plate material; n is the maximum distance from the anchor plate edge to the anchor plate edge; t is the thickness of the anchor plate; $\sigma_{1n}$ is the average compressive stress on the bottom surface of the anchor plate.

Simultaneously, under bending conditions, the bending stress of the anchor plate should meet the requirements of Equations (12) and (13).

(12) $$\sigma_{s}\leq 0.8f_{sy}$$

(13) $$\sigma_{s}=3{\sigma {\left(\frac{n}{t}\right)}^{2}}_{ln}$$

where $f_{sy}$ represents the design value of the tensile strength of the anchor plate, and the calculation results are shown in Table 9.

Based on the calculation results, it can be observed that when the displacement is only up to an inter-story displacement angle of 2.5%, the thickness and strength of the anchor plate meet the requirements. However, during the experimental process, the anchor plate of the WSW-2-46 sample does not meet the requirements when the prestressed steel strand reaches its maximum internal force. In addition, all three specimens have the following deficiencies. First, the diameter of the reserved holes in the concrete bottom beam is much larger than the diameter of the plate holes. Second, the concrete in the installed position of the reserved anchor plate is uneven, resulting in a stress concentration in the compressed area of the plate. As indicated by the experimental observations mentioned above, the anchor plate of the WSW-2-46 specimen was completely damaged, while the anchor plates of the WSW-1-23 and WSW-3-23 samples experienced some degree of deformation.

### 5.5. Analytical Calculation for Bearing Capacity of Specimen

In the experiment, once any of the steel strands, anchor plates, or concrete fails, the specimen loses its load-bearing capacity. Therefore, the stress in the steel strands should not exceed the limits defined by these three aspects.

(14) $$P_{max}=\min(F_{1},F_{2},F_{3})$$

The balance of the moment at the bottom right corner of the wall in Figure 18 yields Equation (15):

(15) $$F\left[h\cdot \cos\alpha +\left(b_{1}+b_{2}\right)\sin\alpha \right]-N\cdot \frac{b}{2}+M\leq \sum P_{i}\cdot b_{i}$$

In the small deformation scenario, where cos⁡α approaches 1 and sin⁡α approaches 0, Equation (15) can be further simplified to Equation (16):

(16) $$Fgh-N\cdot \frac{b}{2}+M\leq \sum P_{i}\cdot b_{i}$$

## 6. Conclusions

This study investigates the lateral resistance performance of prestressed CLT–concrete composite structures with prestressed steel strands. Experimental investigations on three full-scale specimens yield the following key findings:

1. Hysteresis curves of all specimens exhibit an anti-S shape with pinching phenomena, becoming more pronounced with increasing lateral displacement. Skeleton curves show a clear three-stage behavior: elastic, elastic–plastic, and yield stages.

2. Stiffness degradation in prestressed CLT shear walls follows a similar trend, with rapid early-stage degradation and a more gradual decline later. The increase in initial tension on the steel strands improves the initial stiffness, with WSW-2-46 being 1.11 times that of WSW-1-23. The size of the samples has a greater impact on overall stiffness than strand tension, WSW-3-23 being 1.6 times that of WSW-1-23.

3. Energy dissipation occurs in two stages. In the early stage, it results from reduced gaps between the CLT layers and frictional energy dissipation. Once the lateral displacement reaches 20, the steel strands contribute, enhancing energy dissipation. Increased initial tension improves energy dissipation, with WSW-2-46 having 1.7 times the damping coefficient of WSW-1-23. Specimen size impact is significant, with WSW-3-23 having 2.2 times the damping coefficient of WSW-1-23.

4. Concrete–timber structures exhibit a higher maximum load-carrying capacity (25.2% to 28.5%) compared to steel–timber composite structures under identical conditions. The failure modes differ, with timber–steel structures concentrating failure on the nodes, while timber–concrete structures reduce the dependence on steel nodes.

5. Overall failure is primarily governed by the formula in Equation (1). The structural failure is low due to shear wall stiffness, allowing us to treat it as a rigid body. The predominant failure involves local modes: steel strand, anchor plate, and local concrete failures.

## Figures and Tables

**Table 1:** Density determination of standard sample.

Sample ID	Dimensions			Mass (g)	Density (kg/m^3^)	Average Density (kg/m^3^)
	*l*	*b*	*t*			
1	17.2	7.9	7.8	477.50	450.530	445.36
2	17.0	8.0	7.9	450.60	419.397	
3	17.0	7.9	7.8	468.82	447.511	
4	17.2	7.8	7.9	427.41	403.269	
5	17.8	8.0	7.9	445.51	396.023	
6	17.1	7.9	8.0	496.29	459.222	
7	17.2	7.9	7.8	474.02	447.246	
8	17.3	8.0	7.8	466.27	431.923	
9	17.3	7.9	7.8	467.25	438.592	
10	17.0	7.8	8.0	510.20	480.958	
11	17.0	7.9	7.8	503.17	480.335	
12	17.2	7.9	7.9	448.09	417.429	
13	17.0	7.9	7.9	509.32	480.051	
14	17.2	7.9	7.9	453.47	417.429	
15	17.1	8	7.9	484.25	480.051	
16	17.1	7.9	7.8	512.53	422.441	
17	17.3	7.9	7.9	446.02	448.081	
18	17.0	8.0	7.9	514.11	480.282	
19	17.2	7.8	7.9	489.98	413.099	
20	17.2	7.9	7.8	479.17	446.382	
21	17.2	7.8	7.9	455.18	429.470	
22	17.2	7.8	7.9	458.56	438.206	
23	17.1	7.9	7.8	490.95	460.031	
24	17.3	7.8	7.9	487.52	457.325	

**Table 2:** Parameters related to test specimens.

Sample ID	Dimensions (mm)	Number of Layers	Thickness of Each Layer (mm)	Diameter of Steel Strand (mm)	Initial Prestressing Forces (kN)
WSW-1-23	2400 × 1200 × 175	5	35	15.2	23
WSW-2-46	2400 × 1200 × 175	5	35	15.2	46
WSW-3-23	2400 × 2400 × 175	5	35	15.2	23

**Table 3:** Rubber material parameters.

Specimen	Location	Dimension (mm)	Hardness	Elastic Modulus (N/cm^2^)	Shear Modulus (N/cm^2^)	Correction Factor
WSW-1-23	Bottom	1300 × 175 × 10	60	445	106	0.57
WSW-2-23	Side	50 × 175 × 300				
WSW-3-23	Bottom	1300 × 175 × 10				
	Side	50 × 175 × 300				

**Table 4:** Comparison of experimental parameters.

Sample ID	Dimensions (mm)	Materials	Drift Angle (%)	Bottom Beam	Initial Prestressing Forces (kN)	Maximum Load-Carrying Capacity (kN)
PS	1200 × 2200 × 175	Spruce–Pine–Fir	2.5	Steel	23/46	52.5/60.8
PH	1200 × 2200 × 175		3	Steel	N/A	25.2
PQ	1200 × 2400 × 175		2.5	Reinforced concrete	23/46	73.4/81.3

**Table 5:** Elastic parameters of CLT (MPa).

E_L_	E_R_	E_T_	G_LR_	G_LT_	G_RT_	γ_LR_	γ_LT_	γ_RT_
17,930	1520	1130	1345	1075	322	0.43	0.37	0.63

**Table 6:** Verification and error analysis of prestressed CLT shear walls.

	Sample ID	Dimensions (mm)	Number of Steel Strands	Strength Verification Values	Errors (%)
Theoretical values	WSW-1-23	1200 × 2400 × 175	2	0.194	-
	WSW-2-46	1200 × 2400 × 175	2	0.189	-
	WSW-3-23	2400 × 2400 × 175	2	0.203	-
	WSW-1-0	1200 × 2400 × 175	2	0.04	-
	WSW-1-70	1200 × 2400 × 175	2	0.197	-
	WSW-1-90	1200 × 2400 × 175	2	0.201	-
	Aspect ratio = 1.2	2880 × 2400 × 175	2	0.217	-
	Aspect ratio = 1.3	3120 × 2400 × 175	2	0.221	-
	Aspect ratio = 1.4	3360 × 2400 × 175	2	0.226	-
Experimental values	WSW-1-23	1200 × 2400 × 175	2	0.179	7.5
	WSW-2-46	1200 × 2400 × 175	2	0.143	23
	WSW-3-23	2400 × 2400 × 175	2	0.186	8.3
Numerical simulation	WSW-1-23	1200 × 2400 × 175	2	0.183	5.6
	WSW-2-46	1200 × 2400 × 175	2	0.179	5.2
	WSW-3-23	2400 × 2400 × 175	2	0.193	4.8

**Table 7:** Calculation of local compression in the concrete bottom beam.

Sample ID	$F_{2}$ (kN)	$1.3\eta_{s}\beta f_{cd}A_{\ln}/\gamma_{0}$ (kN)
WSW-1-23	295.8	2872.5
WSW-2-46	112.8	
WSW-3-23	321.6	

**Table 8:** Thickness of anchor plate.

Sample ID	Thickness of Anchor Plate (mm)	Maximum Calculation Thickness (mm)	Anchor Plate Thickness Corresponding to Drift Angle of 2.5% (mm)
WSW-1-23	18	13.57	10.35
WSW-2-46	12		10.7
WSW-3-23	18		11.52

**Table 9:** Bending stress of the anchor plate.

Sample ID	Thickness of Anchor Plate (mm)	$0.8f_{sy}$ (MPa)	Stress Corresponding to Drift Angle of 2.5% (mm)	Maximum Calculated Stress (MPa)
WSW-1-23	18	188	31.42	93.2
WSW-2-46	12		78.213	201.5
WSW-3-23	18		88	93.2

## Data Availability

The raw data supporting the conclusions of this article will be made available by the authors on request.
